# Scattering Theory in an N-Pole Semiconductor Quantum Device: The Unitarity of the Current S-Matrix and Current Conservation

**DOI:** 10.3390/mi16030306

**Published:** 2025-03-05

**Authors:** Jan Kučera, Ulrich Wulf, George Alexandru Nemnes

**Affiliations:** 1 Institute of Physics of the Czech Academy of Sciences, Cukrovanická 10/112, 16200 Praha, Czech Republic; kucera@fzu.cz; 2 Faculty 1, Brandenburg University of Technology Cottbus-Senftenberg, Platz der Deutschen Einheit 1, Konrad-Wachsmann-Allee 13, 03046 Cottbus, Germany; 3 Faculty of Physics, University of Bucharest, 077125 Magurele-Ilfov, Romania; nemnes@solid.fizica.unibuc.ro

**Keywords:** S-matrix, Landauer–Büttiker formalism, N-pole device, R-matrix, current conservation

## Abstract

In a number of previous publications, scattering theory for N-pole semiconductor quantum devices was developed. In the framework of the Landauer–Büttiker formalism, an S-matrix was constructed with the aid of an R-matrix providing a mapping of the in-going waves onto the out-going waves. These waves include propagating waves and evanescent waves, the latter of which decay exponentially in the leads which are connected to the active region of the N-pole device. In order to formulate the current conservation in the N-pole device, it is necessary to define the current S-matrix schematically as $\tilde{S}=k^{1/2}Sk^{-1/2}$, where *k* contains the information about the k-vectors of the mentioned in- and out-going waves. In this paper, we show how the complete current S-matrix is calculated including the coupling between the propagating and evanescent components and coupling to the bound states in the active device region. One then finds a sub-matrix of $\tilde{S}$ which is unitary and which is restricted to the space of the propagating components. We demonstrate that current conservation is associated with the unitarity just of this sub-matrix.

## 1. Introduction

**Figure 1:**
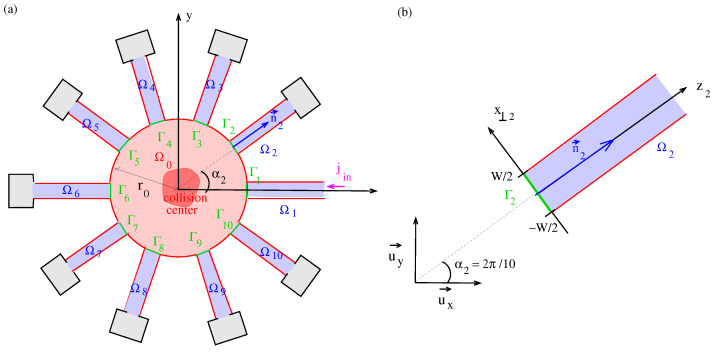
(**a**) N-pole device with a scattering center in $\Omega_{0}$ and supplying leads located in $\Omega_{s}$, s=1,⋯N=10. The scattering process is confined to the scattering domain ${⋃}_{s=0}^{N}\Omega_{s}$ associated with fixed boundary conditions for the wave functions along Γ (the red line). The shown N-pole device we take in Section 5 as an R-matrix model for the measurement of the Heisenberg S-matrix $S(\vec{k},{\vec{k}}^{\prime })$ in a diffraction experiment of a non-relativistic particle beam in a vacuum chamber: There is one incident particle beam in lead s=1. The distribution of the out-going waves can be measured under the nine angles $\alpha_{s}$, s=2,⋯10. (**b**) HE Local coordinates $\left(z_{s},x_{⊥;s}\right)$ in the lead $\Omega_{s}$ for s=2. The local coordinate system is rotated by an angle of $\alpha_{2}=2\pi /10$ with respect to the global coordinate system spanned by the Cartesian unit vectors ${\vec{u}}_{x}$ and ${\vec{u}}_{y}$.

The R-matrix method, developed by Wigner and Eisenbud [1], was initially employed to describe atomic collisions [2,3,4]. Later, the R-matrix formalism became suitable for describing the quantum transport in mesoscopic semiconductor N-pole devices within scattering theory [5], and further developments followed [6,7,8,9]. Examples of the application of this approach include conventional MOSFETs [8], nanowire transistors [10], spin-FETs [11], quantum logic gates [12], SOI transistors [13], and the two-channel transistor as a proposal for a new device architecture based on SOI technology [14]. In our N-pole scattering theory, the active region of the device is considered the scattering center, while the supplying leads represent the asymptotic regions in which the in-going and out-going waves are formed. The whole scattering process is treated within fixed boundary conditions, i.e., the scattering functions vanish outside a restricted scattering domain associated with the semiconductor device (see Figure 1 as an example). This is in contrast to standard scattering theory where the scattering domain is unrestricted and scattering occurs between infinitely extended plane waves (see the monographs [15,16,17] and the original papers on the S-matrix [18,19].

A note on the unitarity: In the works by Heisenberg [18,19] scattering was conceived as a spatially unrestricted multi (at least two)-particle problem. Assuming that the scattering potential depends only on the *relative* coordinates between the particles the Hamilton operator commutes with the total momentum of all the particles which is therefore conserved. Using momentum conservation the unitarity of the S-matrix can be shown (see e.g., Equation (7.101) of Ref. [16]). In the standard approach today the configuration space is usually reduced to the relative coordinates (see Section 1.2 of [16]). In the resulting effective single particle problem in Equation (1.26) of [16] the S-matrix $S(\vec{k},{\vec{k}}^{\prime })$ is non-diagonal, where the wave vectors $\vec{k}$ and ${\vec{k}}^{\prime }$ both refer to the momentum $\vec{p}$ associated with the relative motion. However, $\vec{p}$ does *not* commute with the Hamiltonian of the relative motion in Equation (1.26) of [17]. Therefore it is *not* conserved. The same lack of moment conservation applies to the scattering problem associated with transport in semiconductor devices for which we derive in the R-matrix theory. One important reason for the lack of moment conservation is the restricted scattering geometry.

In R-matrix scattering theory, the in-going and out-going currents point in the direction of the supplying leads since the corresponding wave functions are bound in the transverse directions, with associated quantization of the levels in the transverse direction. In the framework of the Landauer–Büttiker formalism, the S-matrix was constructed providing a mapping of the in-going laterally bound waves onto the out-going laterally bound waves. These waves include propagating waves and evanescent waves, the latter of which decay exponentially in the leads. Evanescent modes arise first because of the coupling to the truly bound states with negative energy eigenvalues. Second, they arise even at positive energies due to scattering processes into the evanescent out-going scattering channels with a highly energetic transverse mode.

In ref. [9], the relation between the S-matrix and the R-matrix was constructed as taking the shape of a Cayley transform, schematically $S=-{\left[1-\left(i/m\right)Rk\right]}^{-1}\left[1+\left(i/m\right)Rk\right]$, where the matrix *k* was associated with the wave vectors of the in- and out-going waves. It is known that the Cayley transform of a matrix is unitary if, and only if, the matrix is skew-Hermitian [20,21]. It is seen that the S-matrix is not unitary because of the symmetry properties of the product Rk. We then define the symmetrical current S-matrix, schematically $\tilde{S}=k^{1/2}Sk^{-1/2}$, which is immediately seen to be unitary if the evanescent modes are neglected. In this paper, we show that one can find a sub-matrix of $\tilde{S}$ (a ‘reduced current S-matrix’) which is unitary if the complete current S-matrix is calculated including the coupling between the propagating and evanescent components, as well as the coupling to the bound states in the active device region. The reduced current S-matrix is the part of $\tilde{S}$ which is restricted to the vector space of the propagating components. We demonstrate that current conservation is associated with just the unitarity of the reduced current S-matrix. The unitarity of the reduced current S-matrix allows us to construct via the Cayley transform a Hermitian current reactance matrix $K=k^{1/2}Rk^{1/2}$ that is structurally analogous to the resonance theory by [22,23,24,25,26] in standard scattering theory with an infinite scattering volume. However, in contrast to standard scattering theory, the R-matrix theory leads to a perturbation series in powers of *K*, with the zero-order term describing the system with total reflection (zero transmission), while the first-order term ∝K describes the transmission resonances.

In the discussion of our results, we relate the presented R-matrix approach to the general definition of the S-matrix $S(\vec{k},\vec{k^{\prime }})$, which connects the infinitely extended in- and out-going plane waves where $\vec{k}$ and ${\vec{k}}^{\prime }$ are the wave vectors of the plane waves. In a simple R-matrix model for the measurement of $S(\vec{k},\vec{k^{\prime }})$, in a diffraction experiment, it is seen that the fixed boundary conditions in the R-matrix theory can be associated with the boundaries of the vacuum chamber in which the experiment is carried out. The laterally bound character of the in- and out-going waves in the R-matrix theory can be associated with the finite opening of the detectors. Originally, Heisenberg introduced the S-matrix to examine experimentally accessible quantities [15]. Here, we remark that if elementary quantum processes are considered, only a few particles can be involved, which necessarily requires vacuum conditions confined to a finite scattering volume, which is represented by the fixed boundary conditions in R-matrix theory. Also, we remark that plane waves with an infinite lateral extension are not measurable. A detector necessarily has a finite opening confining the waves in the perpendicular direction. Therefore, reflections occur at the transition from the vacuum chamber to the leads. This phenomenon is described by the overlap factor $c_{ksn}$ introduced in Equation (44) of [9] in the R-matrix approach.

## 2. The Construction of the N-Pole S-Matrix—The R-Matrix Approach

In ref. [9], we developed R-matrix theory for the potential scattering from a collision center located in a central domain in $\Omega_{0}$ (see Figure 1).

The collision center is closed except for the independent in-coming and out-going waves confined in N-independent leads located in $\Omega_{s}$ with s=1,⋯N. We seek scattering solutions for the time-independent Schrödinger equation

(1) $$\left(-\frac{\hbar^{2}}{2m^{*}}\Delta +V\left(\vec{r}\right)-E\right)\Psi \left(\vec{r},E\right)=0$$

in the restricted scattering domain ${⋃}_{s=0}^{N}\Omega_{s}$. For simplicity of its presentation, we here assume a special potential of the form

(2) $$V\left(\vec{r}\right)=\left\{\begin{array}{cc} \mathrm{arbitrary} & \mathrm{for}\;\;\vec{r}\in \Omega_{0}\\ 0 & \mathrm{for}\;\;\vec{r}\in \Omega_{s},\;s=1,\cdots N\\ \infty & \mathrm{otherwise}. \end{array}\right.$$

These results can be easily transferred to a general case. As in Equation (4) of ref. [9] the asymptotics of the stationary solutions of the Schrödinger equation in $\Omega_{s}$ are given by

(3) $$\Psi \left(\vec{r}\in \Omega_{s},E\right)=\sum_{n}\Psi_{sn}^{in}\exp\left(-ik_{n}z_{s}\right)\Phi_{n}\left(x_{⊥;s}\right)+\sum_{n}\Psi_{sn}^{out}\exp\left(ik_{n}z_{s}\right)\Phi_{n}\left(x_{⊥;s}\right).$$

The first factor on the right-hand side is the in-going part, with the expansion coefficients $\Psi_{sn}^{in}$, and the second factor the out-going part, with the expansion coefficients $\Psi_{sn}^{out}$. While an exact definition of the quantities is given in ref. [9], we illustrate them here in discussing the N=10-pole geometry of the example system in Figure 1. In the two-dimensional geometry, we denote with ${\vec{u}}_{x}$, ${\vec{u}}_{y}$ the Cartesian unit vectors characterizing the global coordinate system $\vec{r}=x\;{\vec{u}}_{x}+y\;{\vec{u}}_{y}$. In each lead, we can define a normal unit vector ${\vec{n}}_{s}$ and the corresponding transverse unit vector ${\vec{n}}_{s}^{⊥}$

(4) $${\vec{n}}_{s}={\vec{u}}_{x}\cos\alpha_{s}+{\vec{u}}_{y}\sin\alpha_{s}\;\;\;\mathrm{and}\;\;\;{\vec{n}}_{s}^{⊥}=-{\vec{u}}_{x}\sin\alpha_{s}+{\vec{u}}_{y}\cos\alpha_{s},$$

with $\alpha_{s}=\left(s-1\right)2\pi /N$. In the local coordinate system in $\Omega_{s}$, the longitudinal coordinate is defined as

(5) $$z_{s}=\vec{r}\cdot {\vec{n}}_{s}-r_{0}=x\cos\left(\alpha_{s}\right)+y\sin\left(\alpha_{s}\right)-r_{0}$$

where $r_{0}$ is the radius of the circular scattering area $\Omega_{0}$. The transverse coordinates are given by

(6) $$x_{⊥;s}=\vec{r}\cdot {\vec{n}}_{s}^{⊥}=-x\sin\left(\alpha_{s}\right)+y\cos\left(\alpha_{s}\right).$$

The transverse mode functions $\Phi_{n}\left(x_{⊥}\right)$ with the index *n* are the solutions of the eigenvalue problem

(7) $$\left[-\frac{\hbar^{2}}{2m^{*}}\frac{d^{2}}{dx_{⊥}^{2}}-E_{n}^{⊥}\right]\;\Phi_{m}\left(x_{⊥}\right)=0.$$

with the fixed boundary conditions $\Phi_{n}\left(x_{⊥}=\pm W/2\right)=0$. Then,

(8) $$\Phi_{n}\left(x_{⊥}\right)=\;\sqrt{\frac{2}{W}}\sin\left[q_{n}\left(x_{⊥}+W/2\right)\right]\;\;\;\mathrm{with}\;\;\;q_{n}=n\frac{\pi }{W},\;\;\;\;\;n\in N$$

and

(9) $$E_{n}^{⊥}=\frac{\hbar^{2}}{2m^{*}}q_{n}^{2}.$$

From energy conservation, the wave numbers of the plane waves in the longitudinal direction are given by

(10) $$k_{n}=\hbar^{-1}\sqrt{2m^{*}\left(E-E_{n}^{⊥}\right)}\equiv \left\{\begin{array}{cc} \kappa_{n} & \;\;\mathrm{for}\;E-E_{n}^{⊥}>0\\ i\kappa_{n} & \;\;\mathrm{for}\;E-E_{n}^{⊥}<0 \end{array}\right.$$

with $\kappa_{n}\geq 0$. In each channel, the longitudinal coordinate $z_{s}\geq 0$ is directed outward. Therefore, the oscillating out-going components resulting at $E-E_{n}^{⊥}>0$ are proportional to $\exp\left(i\kappa_{n}z_{s}\right)$ (‘propagating components’). For $E-E_{n}^{⊥}<0$, the out-going components are exponentially damped $\propto \exp\left(-\kappa_{n}z_{s}\right)$ (‘evanescent components’). The in-going components are either oscillating $\propto \exp\left(-i\kappa_{n}z_{s}\right)$ for $E-E_{n}^{⊥}>0$ or exponentially growing $\propto \exp\left(\kappa_{n}z_{s}\right)$ for $E-E_{n}^{⊥}<0$. Therefore the evanescent components have to be excluded in the in-going part. In ref. [9], we constructed the S-matrix, which was defined as a linear mapping

(11) $$\Psi_{sn}^{out}=S_{sn;s^{\prime }n^{\prime }}\Psi_{s^{\prime }n^{\prime }}^{in}\Leftrightarrow \Psi_{\nu }^{out}=S_{\nu \nu^{\prime }}\Psi_{\nu^{\prime }}^{in}$$

introducing the composite channel index ν=(s,n).

In Equation (29) of [9], we wrote for the S-matrix, ${\left(S\right)}_{\nu \nu^{\prime }}=S_{\nu \nu^{\prime }}$,

(12) $$S=-{\left(1-\frac{i}{m^{*}}Rk\right)}^{-1}\left(1+\frac{i}{m^{*}}Rk\right).$$

Here, the k-matrix is given by

(13) $${\left(k\right)}_{\nu \nu^{\prime }}=\delta_{\nu \nu^{\prime }}k_{n}$$

and the real symmetrical R-matrix is taken from Equation (27) of [9]:

(14) $$R_{\nu \nu^{\prime }}=\int_{\Gamma_{s}}d\Gamma_{s}\;\int_{\Gamma_{s^{\prime }}}d\Gamma_{s^{\prime }}^{\prime }\;\Phi_{\nu }\left({\vec{r}}_{⊥;s}\right)\Phi_{\nu^{\prime }}\left({\vec{r}}_{⊥;s^{\prime }}^{\prime }\right)R\left(\vec{r},{\vec{r}}^{\prime };E\right)=-\frac{\hbar^{2}}{2}\sum_{l=1}^{\infty }\frac{\chi_{l\nu }\chi_{l\nu^{\prime }}}{E-E_{l}}.$$

The Wigner–Eisenbud functions $\chi_{l}$ are the solutions of the Schrödinger equation

(15) $$\left[-\frac{\hbar^{2}}{2m^{*}}\Delta +V\left(\vec{r}\right)-E_{l}\right]\;\chi_{l}\left(\vec{r}\right)=0$$

in the domain $\Omega_{0}$. Here, one imposes Wigner–Eisenbud boundary conditions, i.e., Neumann boundary conditions of the vanishing normal derivative on $\Gamma_{s}$,

(16) $$\frac{\partial \chi_{l}}{\partial {\vec{n}}_{s}}=0\;\;\;\;for\;\;\;\;\;\vec{r}\in \Gamma_{s}$$

and Dirichlet boundary conditions on the remaining surface of $\Omega_{0}$, denoted by $\partial \Omega_{0}$, writing

(17) $$\chi_{l}=0\;\;\;\;for\;\;\;\;\;\vec{r}\in \partial \Omega_{0}.$$

We then introduce

(18) $$\chi_{l\nu }=\int_{\Gamma_{s}}d\Gamma_{s}\;\Phi_{\nu }\left({\vec{r}}_{⊥;s}\right)\chi_{l}\left(\vec{r}\right).$$

The minus sign in Equation (12) can easily be understood as the limit of total reflection, in our example setting in Equation (2) $V(\vec{r}\in \Omega_{0})\to \infty$. Then, $E_{l}\to \infty$, and according to (14), R→0 so that S→−1. From (11), we then have $\Psi_{\nu }^{out}=-\Psi_{\nu }^{in}$ corresponding to standing waves. We also introduce the symmetrical current S-matrix

(19) $$\begin{array}{ccc} \tilde{S} & = & k^{1/2}Sk^{-1/2}=-k^{1/2}{\left(1-\frac{i}{m^{*}}Rk\right)}^{-1}\left(1+\frac{i}{m^{*}}Rk\right)k^{-1/2}\\ & = & -k^{1/2}{\left(1-\frac{i}{m^{*}}Rk\right)}^{-1}k^{-1/2}k^{1/2}\left(1+\frac{i}{m^{*}}Rk\right)k^{-1/2}\\ & = & -\left[k^{1/2}{\left(1-\frac{i}{m^{*}}Rk\right)}^{-1}k^{-1/2}\right]\left[k^{1/2}\left(1+\frac{i}{m^{*}}Rk\right)k^{-1/2}\right]\\ & = & -{\left[k^{1/2}k^{-1/2}-i\Omega \right]}^{-1}\left[\left(k^{1/2}\right)k^{-1/2}+i\Omega \right]\\ & = & -{\left(1-i\Omega \right)}^{-1}\left(1+i\Omega \right). \end{array}$$

with the symmetrical current R-matrix

(20) $$\Omega_{\nu \nu^{\prime }}=\frac{1}{m^{*}}{\left(k_{\nu }\right)}^{1/2}R_{\nu \nu^{\prime }}{\left(k_{\nu^{\prime }}\right)}^{1/2}.$$

In (19), we exploited the fact that for three square matrices, one has ${\left(ABC\right)}^{-1}=C^{-1}B^{-1}A^{-1}$.

We number the components ν↔j so that for j=1,⋯N, the wave number $k_{\nu }$ is real, and for the remaining ones, $k_{\nu }$ is imaginary. One obtains a diagonal matrix

(21) $$k=\left[\begin{array}{ccccc} \kappa_{1} & \; & & \; & 0\\ \; & \ddots & \; & & \\ \; & \; & \kappa_{N} & \; & \\ \; & \; & & i\kappa_{N+1} & \\ 0 & \; & & \; & \ddots \end{array}\right]\;\;\;\;\mathrm{or}\;\;\;\;{\left(k\right)}_{jj^{\prime }}=\delta_{jj^{\prime }}k_{\nu }.$$

Furthermore, we have

(22) $$k^{1/2}=\left[\begin{array}{ccccc} \kappa_{1}^{1/2} & \; & & \; & 0\\ \; & \ddots & \; & & \\ \; & \; & \kappa_{N}^{1/2} & \; & \\ \; & \; & & \frac{1+i}{\sqrt{2}}\kappa_{N+1}^{1/2} & \\ 0 & \; & & \; & \ddots \end{array}\right]\;\;\;\mathrm{and}\;\;\;k^{-1/2}=\left[\begin{array}{ccccc} \kappa_{1}^{-1/2} & \; & & \; & 0\\ \; & \ddots & \; & & \\ \; & \; & \kappa_{N}^{-1/2} & \; & \\ \; & \; & & \frac{1-i}{\sqrt{2}}\kappa_{N+1}^{-1/2} & \\ 0 & \; & & \; & \ddots \end{array}\right].$$

Using the Heaviside function Θ, we can rewrite (20) into the form

(23) $${\left(\Omega \right)}_{jj^{\prime }}=\kappa_{j}^{1/2}\kappa_{j^{\prime }}^{1/2}R_{jj^{\prime }}i^{\;\Theta (j-N)/2}\;\;i^{\;\Theta (j^{\prime }-N)/2}={\bar{\Omega }}_{jj^{\prime }}i^{\;\Theta (j-N)/2}\;\;i^{\;\Theta (j^{\prime }-N)/2}$$

with the real symmetrical matrix ${\bar{\Omega }}_{jj^{\prime }}=\kappa_{j}^{1/2}\kappa_{j^{\prime }}^{1/2}R_{jj^{\prime }}$ and Θ(0)≡0.

## 3. Current Conservation

As can be taken from (A5)–(A11) in ref. [9], the absolute value of the current associated with a single propagating component $\Psi_{j}^{in/out}\exp\left(\pm ik_{j}z_{s}\right)\Phi_{j}\left({\vec{r}}_{⊥;s}\right)$ in (3) is given by

(24) $$I_{j\leq N}^{in/out}=\frac{\hbar }{m^{*}}k_{j}{\left|\Psi_{j}^{in/out}\right|}^{2}\equiv \frac{\hbar }{m^{*}}{\left|\psi_{j}^{in/out}\right|}^{2}$$

with the coefficient $\psi_{j}^{in/out}=k_{j}^{1/2}\Psi_{j}^{in/out}$ for j≤N. From (11), we obtain with $\psi_{J>N}^{in}=0$

(25) $$\psi_{j\leq N}^{out}=\sum_{j^{\prime }=1}^{N}k_{j}^{1/2}S_{jj^{\prime }}k_{j^{\prime }}^{-1/2}\psi_{j^{\prime }}^{in}=\sum_{j^{\prime }=1}^{N}\Sigma_{jj^{\prime }}\psi_{j^{\prime }}^{in}$$

with the N×N reduced current S-matrix Σ

(26) $$\Sigma_{jj^{\prime }}=k_{j}^{1/2}S_{jj^{\prime }}k_{j^{\prime }}^{-1/2}\;\;j,j^{\prime }=1,\cdots N.$$

The total charge current is then given by

(27) $$\begin{array}{ccc} I^{out} & = & \sum_{j=1}^{N}I_{j}^{out}=\frac{e\hbar }{m^{*}}\sum_{j=1}^{N}k_{j}{\left(\psi_{j}^{out}\right)}^{*}\psi_{j}^{out}=\frac{e\hbar }{m^{*}}\sum_{jj^{\prime }j^{\prime \prime }=1}^{N}\Sigma_{jj^{\prime }}^{*}\Sigma_{jj^{\prime \prime }}{\left(\psi_{j^{\prime }}^{in}\right)}^{*}\psi_{j^{\prime \prime }}^{in}\\ & = & \frac{e\hbar }{m^{*}}\sum_{jj^{\prime }j^{\prime \prime }=1}^{N}\Sigma_{j^{\prime }j}^{†}\Sigma_{jj^{\prime \prime }}{\left(\psi_{j^{\prime }}^{in}\right)}^{*}\psi_{j^{\prime \prime }}^{in}. \end{array}$$

Current conservation results if Σ is unitary:

(28) $$\Sigma_{j^{\prime }j}^{-1}=\Sigma_{jj^{\prime }}^{†}.$$

Then,

(29) $$\begin{array}{ccc} I^{out} & = & \frac{e\hbar }{m^{*}}\sum_{jj^{\prime }j^{\prime \prime }=1}^{N}\Sigma_{j^{\prime }j}^{-1}\Sigma_{jj^{\prime \prime }}{\left(\psi_{j^{\prime }}^{in}\right)}^{*}\psi_{j^{\prime \prime }}^{in}=\frac{e\hbar }{m^{*}}\sum_{j}{\left|\psi_{j}^{in}\right|}^{2}=I^{in}. \end{array}$$

In the next section, we show that the reduced current S-matrix Σ is indeed unitary.

## 4. The Unitarity of the Reduced Current S-Matrix

We compare Equations (12) and (19) to the similar Equations (7.59) and (7.60) for standard scattering theory (an unrestricted scattering domain) from ref. [19] for the S-matrix

(30) $$S=\left(1+iK\right){\left(1-iK\right)}^{-1}$$

and the Hermitian reactance matrix

(31) $$K^{†}=K.$$

All three Equations (12), (19), and (30) for the current S-matrix can be cast as a Cayley transform (obviously the order of the matrices on r.h.s. of Equation (32) is irrelevant, see [21])

(32) $$F=\left(1-A\right){\left(1+A\right)}^{-1}$$

setting F=S to obtain (30), −S to obtain (12), and $-\tilde{S}$ to obtain (19). It has been proven by Cayley [20] that *A* is skew-Hermitian if and only if F is unitary with −1 not being an eigenvalue of *F*. For the S-matrix in standard scattering theory, we read off A=−iK in Equation (30) so that *A* is skew-Hermitian and S is unitary. From Equation (19), it is seen that $A=-iRk/m^{*}$ in the R-matrix approach. Since *R* is symmetrical, the matrix Rk is asymmetrical in such a way that *A* cannot have an anti-symmetrical real part and a symmetrical imaginary part of its compounds, $A^{H}\neq -A$. Thus, the S-matrix in R-matrix theory for a restricted system geometry is not unitary.

For the current S-matrix (19), we have

(33) $$\begin{array}{ccc} A_{jj^{\prime }} & = & -i\Omega_{jj^{\prime }}=-i{\bar{\Omega }}_{jj^{\prime }}i^{\Theta (j-N)/2}i^{\Theta (j^{\prime }-N)/2}\\ & = & {\bar{\Omega }}_{jj^{\prime }}\times \left\{\begin{array}{cc} -i & \mathrm{for}\;\;j^{\prime },j\leq N\\ 1 & \mathrm{for}\;\;j^{\prime },j>N\\ \frac{1-i}{\sqrt{2}} & \mathrm{for}\;\;j^{\prime }\leq N,j>N\;\;\mathrm{or}\;j^{\prime }>N,j\leq N \end{array}\right. \end{array}$$

and $\tilde{S}$ is seen to be symmetrical. We find

(34) $$A_{jj^{\prime }}^{*}={\bar{\Omega }}_{jj^{\prime }}\times \left\{\begin{array}{cc} i & \mathrm{for}\;\;j^{\prime },j\leq N\\ 1 & \mathrm{for}\;\;j^{\prime },j>N\\ \frac{1+i}{\sqrt{2}}\; & \mathrm{for}\;\;j^{\prime }\leq N,j>N\;\;\mathrm{or}\;j^{\prime }>N,j\leq N. \end{array}\right.$$

It is found that *A* is not skew-Hermitian and thus $\tilde{S}$ is not unitary either.

However, from Equation (29), it is seen that current conservation only requires that the N×N matrix reduced current S-matrix Σ has to be unitary. To show the unitarity of the reduced current S-matrix, we arrange *A* into a block matrix

(35) $$A=\left[\begin{array}{cc} A^{11} & A^{12}\\ A^{21} & A^{22} \end{array}\right]=\left[\begin{array}{cc} \hat{P} & \hat{W}\\ {\hat{W}}^{T} & \hat{H} \end{array}\right]\;,$$

with a skew-Hermitian part

(36) $${\hat{P}}_{jj^{\prime }}=-{\hat{P}}_{j^{\prime }j}^{*}=-i{\bar{\Omega }}_{jj^{\prime }}\;\;\;\;\mathrm{for}\;\;j^{\prime },j\leq N$$

acting only on the propagating modes, a Hermitian part

(37) $${\hat{H}}_{jj^{\prime }}={\hat{H}}_{j^{\prime }j}^{*}={\bar{\Omega }}_{jj^{\prime }}\;\;\;\;\mathrm{for}\;\;\;\;\mathrm{for}\;\;j^{\prime },j>N$$

acting solely on the evanescent modes, and a mixed Hermitian and skew-Hermitian matrix

(38) $${\hat{W}}_{jj^{\prime }}=\frac{1-i}{\sqrt{2}}{\bar{\Omega }}_{jj^{\prime }}\;\;\;\;\mathrm{for}\;\;\;\;\mathrm{or}\;\;\;\;j^{\prime }\leq N,j>N\;\;or\;j^{\prime }>N,j\leq N$$

representing the interaction between the propagating and evanescent modes. Here, we emphasize that only for $\hat{W}=0$ is the vector space separated into a propagating part with a skew-Hermitian matrix $\hat{P}$ and an evanescent part with a Hermitian matrix $\hat{H}$. However, in the general case, one has $\hat{W}\neq 0$, even without the existence of bound states. Without bound states, a finite matrix $\hat{W}$ arises because of the inevitable coupling to higher transverse modes with j>N. Setting $F=-\tilde{S}$ in Equation (32), we write

(39) $$\tilde{S}=-{\left[\begin{array}{cc} 1+\hat{P} & \hat{W}\\ {\hat{W}}^{T} & 1+\hat{H} \end{array}\right]}^{-1}\left[\begin{array}{cc} 1-\hat{P} & -\hat{W}\\ -{\hat{W}}^{T} & 1-\hat{H} \end{array}\right]\;.$$

To invert the first factor on the r. h. s. of (39), we introduce for brevity $Q={\left[1+\hat{P}-\hat{W}{\left(1+\hat{H}\right)}^{-1}{\hat{W}}^{T}\right]}^{-1}$. Now using Equation (A8), we observe that

(40) $$\begin{array}{ccc} & & {\left[\begin{array}{cc} 1+\hat{P} & \hat{W}\\ {\hat{W}}^{T} & 1+\hat{H} \end{array}\right]}^{-1}\\ & & =\;\left[\begin{array}{cc} Q & -Q\hat{W}{\left(1+\hat{H}\right)}^{-1}\\ -{\left(1+\hat{H}\right)}^{-1}{\hat{W}}^{T}Q & \;{\left(1+\hat{H}\right)}^{-1}+{\left(1+\hat{H}\right)}^{-1}{\hat{W}}^{T}Q\hat{W}{\left(1+\hat{H}\right)}^{-1} \end{array}\right].\; \end{array}$$

We can now find the reduced current S-matrix defined in (26):

(41) $$\begin{array}{ccc} \Sigma & = & -\left[\begin{array}{cc} Q & \;-Q\hat{W}{\left(1+\hat{H}\right)}^{-1} \end{array}\right]\left[\begin{array}{c} 1-\hat{P}\\ -{\hat{W}}^{T} \end{array}\right]\\ & = & -Q\left(1-\hat{P}\right)+Q\hat{W}{\left(1+\hat{H}\right)}^{-1}{\hat{W}}^{T}\\ & = & -{\left[1+\hat{P}-\hat{W}{\left(1+\hat{H}\right)}^{-1}{\hat{W}}^{T}\right]}^{-1}\left[1-\hat{P}+\hat{W}{\left(1+\hat{H}\right)}^{-1}{\hat{W}}^{T}\right]\\ & = & -{\left(1-i\hat{K}\right)}^{-1}\left(1+i\hat{K}\right)\\ & = & -1+2\hat{T}, \end{array}$$

In the last step of (41), we have introduced the transition matrix

(42) $$\hat{T}=-\frac{i\hat{K}}{1-i\hat{K}}=-\sum_{n=1}^{\infty }{\left(i\hat{K}\right)}^{n}$$

as described in detail in the context of Equation (9) of [24]. Comparing the second-last line of (41) with (30), we define the current reactance matrix

(43) $$\hat{K}=i\left[\hat{P}-\hat{W}{\left(1+\hat{H}\right)}^{-1}{\hat{W}}^{T}\right]={\bar{\Omega }}^{11}-{\bar{\Omega }}^{12}{\left(1+{\bar{\Omega }}^{22}\right)}^{-1}{\bar{\Omega }}^{21}$$

which is real and symmetrical. It follows that Σ is unitary, from which, in turn, current conservation follows according to Equation (29). Equation (43) shows that $\hat{K}$ reduces into the current R-matrix if one neglects yjr evanescent components in the scattering states. The second term on the r. h. s. of (43) gives the correction resulting from the coupling to the evanescent components so that $\hat{K}$ can be regarded as a corrected current R-matrix. The power series in (42) can be interpreted as a perturbation series for the current S-matrix in R-matrix theory. However, in contrast to the perturbation series for S in standard scattering theory resulting from the Lippmann–Schwinger equation, the zero-order term in (42) represents a closed system, i.e., total reflection. In the standard scattering theory, for a system with a unrestricted scattering domain, the zero-order term represents the complementary limit of an open system with perfect transmission. As a consequence, the first-order correction in standard scattering theory is valid for weak scattering, whereas in the R-matrix theory, the first-order correction represents resonant tunneling transmission resonances.

Since $\hat{K}$ is real and symmetrical, it has *N* real eigenvalues $K_{j}$ and a complete orthonormal system of associated *N*-component real eigenvectors ${\vec{\kappa }}_{j}$ so that

(44) $$\hat{K}=\sum_{j=1}^{N}K_{j}{\vec{\kappa }}_{j}⊗{\vec{\kappa }}_{j}^{T}.$$

(45) $$\Sigma =\sum_{j=1}^{N}\frac{1+iK_{j}}{1-iK_{j}}{\vec{\kappa }}_{j}⊗{\vec{\kappa }}_{j}^{T}=\sum_{j=1}^{N}e^{i2\phi_{j}}{\vec{\alpha }}_{j}⊗{\vec{\alpha }}_{j}^{T}$$

where we introduce the real scattering phases by

(46) $$\frac{1+iK_{j}}{1-iK_{j}}\equiv e^{i2\phi_{j}}\;\;\;\;\to \phi_{j}=\arctan K_{j}.$$

## 5. Discussion

The N-pole device sketched in Figure 1 has the essential features of the Rutherford scattering experiment carried out by Geiger and Marsden in ref. [27], which was carried out in a vacuum chamber and which we represent using the scattering domain ${⋃}_{s=0}^{N}\Omega_{s}$. The rotatable circular platform in Figure 1 of [27] that contains the detectors consisting of the microscope M and the zinc sulfide screen S is replaced with *N* stationary Landauer–Büttiker contacts. The collimation width of the incident particle beam coming from the α-source R and the collimation width of the scattered beam in the zinc sulfide detector to define the scattering angle ϕ are replaced by the width *W* of the leads in $\Omega_{s=1\cdots N}$. In the classical solution of a potential scattering problem, Rutherford calculated the measured particle flux in relation to the incident particle flux, obtaining the well-known $1/\sin\phi^{4}$ [28] dependence of the cross-section, which was confirmed by the experiments in [27]. In our theory, the particle fluxes are related naturally by the current S-matrix.

We now make contact with the S-matrix $S(\vec{k},{\vec{k}}^{\prime })$ in standard scattering theory for an unrestricted scattering domain. We introduce the plane waves in the direction of ${\vec{n}}_{s}$ given by

(47) $$\exp\left(i{\vec{k}}_{sn}\vec{r}\right)=\exp\left(ik_{n}{\vec{n}}_{s}\vec{r}\right)=\exp\left(ik_{n}r_{0}\right)\exp\left(ik_{n}z_{s}\right).$$

extending Equation (10) to define

(48) $${\vec{k}}_{sn}\equiv k_{n}{\vec{n}}_{s}.$$

In the last step of (47), we use Equation (5). We further introduce

(49) $${\bar{\Psi }}_{sn}^{in}=\Psi_{sn}^{in}\exp\left(ik_{n}r_{0}\right)\;\;\;\mathrm{and}\;\;{\bar{\Psi }}_{sn}^{out}=\Psi_{sn}^{out}\exp\left(-ik_{n}r_{0}\right)$$

so that Equation (3) becomes

(50) $$\Psi \left(\vec{r}\in \Omega_{s},E\right)=\sum_{n}{\bar{\Psi }}_{sn}^{in}\exp\left(-i{\vec{k}}_{sn}\vec{r}\right)\Phi_{n}\left(x_{⊥;s}\right)+\sum_{n}{\bar{\Psi }}_{sn}^{out}\exp\left(i{\vec{k}}_{sn}\vec{r}\right)\Phi_{n}\left(x_{⊥;s}\right).$$

The factors $\Phi_{n}\left(x_{⊥;s}\right)$ add a bound component in the transverse direction to the plane waves $\exp\left(\pm i{\vec{k}}_{sn}\vec{r}\right)$, which represent the transverse modes in the detector with an opening of *W*. Equation (48) establishes a one-to-one correspondence between the wave vector of the plane wave and the index pair (sn). Therefore, we can define ${\vec{k}}_{sn}\equiv \vec{K}$ and ${\vec{k}}_{s^{\prime }n^{\prime }}\equiv {\vec{K}}^{\prime }$, as well as ${\bar{\Psi }}_{sn}^{in/out}\equiv {\bar{\Psi }}^{in/out}\left(\vec{K}\right)$. One now obtains from (11)

(51) $$\begin{array}{ccc} & & \Psi_{sn}^{out}=\sum_{s^{\prime }n^{\prime }}S_{sn;s^{\prime }n^{\prime }}\Psi_{s^{\prime }n^{\prime }}^{in}\\ & \Leftrightarrow & {\bar{\Psi }}_{sn}^{out}=\sum_{s^{\prime }n^{\prime }}\exp\left(-ik_{n}r_{0}\right)S_{sn;s^{\prime }n^{\prime }}\exp\left(-ik_{n^{\prime }}r_{0}\right){\bar{\Psi }}_{s^{\prime }n^{\prime }}^{in}\\ & \Leftrightarrow & {\bar{\Psi }}^{out}\left(\vec{K}\right)=\sum_{{\vec{K}}^{\prime }}S\left(\vec{K},{\vec{K}}^{\prime }\right){\bar{\Psi }}^{in}\left({\vec{K}}^{\prime }\right) \end{array}$$

with

(52) $$S\left(\vec{K},{\vec{K}}^{\prime }\right)\equiv S_{sn;s^{\prime }n^{\prime }}\exp\left[-i\left(k_{n^{\prime }}+k_{n}\right)r_{0}\right].$$

## 6. Conclusions

In this article, we first introduce the model setup for a multiterminal, two-dimensional device suitable for a Landauer–Büttiker transport calculation. The key element for this type of a model is the S-matrix. We then introduce the R-matrix and show how the former can be expressed in terms of the latter. Further, we discuss our theory from the perspective of traditional free-space scattering theory, as introduced by Heisenberg. In his approach, the S-matrix is by definiton unitary (see the note on the unitary in the Introduction), and this is considered a central feature of this kind of scattering theory. With the help of the symmetrical current R-matrix (20), we show that while the S-matrix in Equations (11) and (12) itself is not unitary, for our model, the reduced current-S-matrix Σ in Equation (26) is [see Equation (41)]. Equation (41) is the main result of the present article.

Further, we show how Σ is, through the R-matrix, connected to the transition matrix (42), which provides a perturbation series and a basis for the description of resonant tunneling.

## Data Availability

No new data were created or analyzed in this study. Data sharing is not applicable to this article.

## Abbreviations

The following abbreviations are used in this manuscript:

SOI	silicon-on-insulator

## Appendix A. Block Matrix Inversion

In order to express Equation (39) in terms of its block components, we need to know how a block matrix can be inverted. We present the following just for the reader’s convenience; for related information, we refer to [29] and the references therein. Of the procedures that lead to equivalent but different formulae, we chose one that yields the result we need directly. Let us define

(A1) $$M^{-1}\;=\;\left[\begin{array}{cc} a & b\\ c & d \end{array}\right]\;=\;{\left[\begin{array}{cc} A & B\\ C & D \end{array}\right]}^{-1}$$

where we assume that the block *D* can be inverted. Having in mind the properties of the individual block matrices, one can reduce the matrix into the block-diagonal form by performing Gauß elimination first by row (left)

(A2) $$\left[\begin{array}{cc} 1 & -BD^{-1}\\ 0 & 1 \end{array}\right]\left[\begin{array}{cc} A & B\\ C & D \end{array}\right]\;=\;\left[\begin{array}{cc} A-BD^{-1}C & 0\\ C & D \end{array}\right]$$

and then by column (right)

(A3) $$\left[\begin{array}{cc} A-BD^{-1}C & 0\\ C & D \end{array}\right]\left[\begin{array}{cc} 1 & 0\\ -D^{-1}C & 1 \end{array}\right]\;=\;\left[\begin{array}{cc} A-BD^{-1}C & 0\\ 0 & D \end{array}\right]\;.$$

Thus, we have

(A4) $$\left[\begin{array}{cc} 1 & -BD^{-1}\\ 0 & 1 \end{array}\right]\left[\begin{array}{cc} A & B\\ C & D \end{array}\right]\left[\begin{array}{cc} 1 & 0\\ -D^{-1}C & 1 \end{array}\right]\;=\;\left[\begin{array}{cc} A-BD^{-1}C & 0\\ 0 & D \end{array}\right]\;.$$

Using the fact that the triangular matrices can be inverted easily

(A5) $$\left[\begin{array}{cc} 1 & \pm BD^{-1}\\ 0 & 1 \end{array}\right]\left[\begin{array}{cc} 1 & \mp BD^{-1}\\ 0 & 1 \end{array}\right]=1\;,\left[\begin{array}{cc} 1 & 0\\ \mp D^{-1}C & 1 \end{array}\right]\left[\begin{array}{cc} 1 & 0\\ \pm D^{-1}C & 1 \end{array}\right]=1\;,$$

we receive the block-UL expression for the matrix M:

(A6) $$\left[\begin{array}{cc} A & B\\ C & D \end{array}\right]\;=\;\left[\begin{array}{cc} 1 & BD^{-1}\\ 0 & 1 \end{array}\right]\left[\begin{array}{cc} A-BD^{-1}C & 0\\ 0 & D \end{array}\right]\left[\begin{array}{cc} 1 & 0\\ D^{-1}C & 1 \end{array}\right]\;.$$

The block-inversion of the above expression yields

(A7) $$\left[\begin{array}{cc} a & b\\ c & d \end{array}\right]\;=\;\left[\begin{array}{cc} 1 & 0\\ -D^{-1}C & 1 \end{array}\right]\left[\begin{array}{cc} {\left(A-BD^{-1}C\right)}^{-1} & 0\\ 0 & D^{-1} \end{array}\right]\left[\begin{array}{cc} 1 & -BD^{-1}\\ 0 & 1 \end{array}\right]\;.$$

After evaluation of the r.h.s., we finally obtain the desired result

(A8) $$\begin{array}{cc} M^{-1} & =\left[\begin{array}{cc} a & b\\ c & d \end{array}\right] \end{array}$$

$$\begin{array}{ccc} & & =\left[\begin{array}{cc} {\left(A-BD^{-1}C\right)}^{-1} & -{\left(A-BD^{-1}C\right)}^{-1}BD^{-1}\\ {-D^{-1}C\left(A-BD^{-1}C\right)}^{-1} & D^{-1}C{\left(A-BD^{-1}C\right)}^{-1}BD^{-1}+D^{-1} \end{array}\right] \end{array}$$

which is identical to formula (2.3) of Ref. [29].
